# Patients Prefer Boarding in Inpatient Hallways: Correlation with the National Emergency Department Overcrowding Score

**DOI:** 10.1155/2011/840459

**Published:** 2011-12-26

**Authors:** John R. Richards, Gal Ozery, Mark Notash, Peter E. Sokolove, Robert W. Derlet, Edward A. Panacek

**Affiliations:** Department of Emergency Medicine, U.C. Davis Medical Center, Sacramento, CA 95817, USA

## Abstract

*Objective*. The boarding of patients in Emergency Department (ED) hallways when no inpatient beds are available is a major cause of ED crowding. One solution is to board admitted patients in an inpatient rather than ED hallway. We surveyed patients to determine their preference and correlated their responses to real-time National Emergency Department Overcrowding Score (NEDOCS). *Methods*. This was a survey of admitted patients in the ED of an urban university level I trauma center serving a community of 5 million about their personal preferences regarding boarding. Real-time NEDOCS was calculated at the time each survey was conducted. *Results*. 99 total surveys were completed during October 2010, 42 (42%) patients preferred to be boarded in an inpatient hallway, 33 (33%) preferred the ED hallway, and 24 (24%) had no preference. Mean (±SD) NEDOCS (range 0–200) was 136 ± 46 for patients preferring inpatient boarding, 112 ± 39 for ED boarding, and 119 ± 43 without preference. Male patients preferred inpatient hallway boarding significantly more than females. Preference for inpatient boarding was associated with a significantly higher NEDOCS. *Conclusions*. In this survey study, patients prefer inpatient hallway boarding when the hospital is at or above capacity. Males prefer inpatient hallway boarding more than females. The preference for inpatient hallway boarding increases as the ED becomes more crowded.

## 1. Introduction

Emergency Department (ED) crowding, or access block, remains a serious problem worldwide, and its causes are multifactorial [1]. One major problem contributing to ED crowding is lack of inpatient beds and boarding of admitted patients in the ED. This obviates the ability to see new patients in the ED, increases wait and length of stay time, and leads to patient and staff dissatisfaction [2]. Emergency Department boarding also compromises patient safety, as ED physicians and nurses must care for these admitted patients while attending to acutely ill and injured patients in the ED under suboptimal conditions [3–5]. One solution to the problem of boarding patients in the ED is the Full Capacity Protocol developed by Viccellio and colleagues at Stony Brook University Hospital in New York [2, 6]. This protocol mandates the boarding of patients in inpatient hallways (IH) during periods of ED crowding and has been successfully implemented in several hospitals in the United States and Canada [6, 7]. We surveyed ED patients who were admitted and awaiting an inpatient bed about their preferences on boarding in IH, ED hallways (EDH), or having no preference (NP), and correlated these responses with their real-time National Emergency Department Overcrowding Score (NEDOCS). Our hypothesis was patients would prefer IH over EDH, and this preference would be influenced by a higher NEDOCS.

## 2. Materials and Methods

This prospective cross-sectional survey study was performed during the month of October, 2010. Surveys were performed during all days of the week and all hours of the day and night. The study site was an academic ED serving as a level one trauma center to a surrounding population of 5 million. The ED volume is approximately 70,000 patient visits per year. This hospital currently does not implement IH boarding when the hospital is at capacity and the ED is crowded. Patients who were admitted to the hospital and awaiting inpatient beds were identified and asked by trained research associates if they wished to participate in the survey. If patients answered affirmatively, informed consent was obtained first. The survey was then administered by the research associate who documented the patient's answers and ensured completion of all questions and objective data. Demographic information was first obtained and included: age, gender, and race. The patient's location, duration of time in the ED, and history of prior admissions to any hospital were recorded. Duration was time of arrival to the ED to time of survey administration. Satisfaction of care received in the ED was also queried on a 5-point Likert scale. The actual questions from the survey are summarized in Table 1.

Real-time NEDOCS was calculated upon completion of the survey and reflected the actual degree of crowding present during each survey [8]. Some surveys were collected during periods of normal ED volume, and others during severe crowding. Variables collected for this calculation included: (1) total patients (number of total patients in the ED at the time the score is calculated. This includes all patients in all areas including the waiting room, hallways, and fast track); (2) ED beds (total number of ED beds including hallways, chairs, fast track, and other beds that can be used to serve patients at the time the score is calculated); (3) admits (number of admitted patients in the ED at the time the score is calculated); (4) hospital beds (total number of licensed hospital beds); (5) ventilators (number of patients on ventilators/respirators in the ED at the time the score is calculated); (6) longest admit (the longest time a patient has waited for an inpatient bed at the time the score was calculated); (7) last bed time (the wait time from arrival to ED bed for the last patient called). The 200 point NEDOCS scale ranges from 0 to 50 (normal), 51–100 (busy), 101–140 (overcrowded), 141–180 (severe), and above 180 (disaster).

Data were entered into a database and analyzed using STATA (StataCorp LP, College Station, TX). Categorical data were compared with *χ* ^2^ (Chi-square test), and quantitative data with ANOVA and Spearman Rank Correlation. A subanalysis was also performed comparing IH preference to EDH and NP combined and analyzed with Student's *t*-test, *χ* ^2^ test, and multivariate logistic regression. Data are reported as mean ± standard deviation (SD), 95% confidence intervals (CI), unless otherwise stated. Statistical significance is assumed at *P* ≤ 0.05. This study was approved by our hospital's Institutional Review Board.

## 3. Results

A total of 110 patients were approached to participate in the study, and 99 patients consented. Data is presented in Table 2. Of those surveyed, 42 (42%) preferred to be boarded in an IH while awaiting a hospital bed, 33 (33%) preferred the EDH, and 24 (24%) had NP (*P* = 0.02, *χ* ^2^ = 7.4). The average age was 52.3 ± 16.8 years with a range of 19–90. There was no significant difference in age between the IH, EDH, and NP groups. With regard to gender, males preferred IH over EDH and NP combined (*P* = 0.05, *χ* ^2^ = 3.8). The majority of patients surveyed were White (*n* = 45, 45%), and there were no significant differences in boarding preferences between White, Black, Hispanic, and Asian. The location in which patients were surveyed, their duration in the ED prior to being surveyed, and having been admitted to the hospital before did not appear to influence boarding preference. Patients were also asked the reason(s) for their preference (Tables 3 and 4) and suggestions for improving ED crowding (Table 5).

Overall, patients were very satisfied with their medical care while in the ED (4.6 ± 0.8), and there were no significant differences in satisfaction scores for each group and boarding preferences. Correlation between higher satisfaction with their overall care in the ED and lower NEDOCS was observed but did not reach statistical significance (Spearman Rank Correlation *ρ* = −0.14, *P* = 0.08). This was also true with regard to higher satisfaction and shorter ED duration prior to taking the survey (*ρ* = −0.15, *P* = 0.07). Average NEDOCS for the entire group was 124 ± 44, and preference for IH was associated with a significantly higher NEDOCS when compared to EDH and NP separately (*P* = 0.05, ANOVA) as well as combined (*P* = 0.04, Student's *t*-test). Multivariate logistic regression analysis confirmed the aforementioned results, with male gender (OR = 2.2; 95% CI: 1.0, 5.0; *P* = 0.02) and higher NEDOCS (OR = 1.2; 95% CI: 0.8, 1.8; *P* = 0.03) associated with IH preference.

## 4. Discussion

In a survey study of admitted ED patients by Garson and associates, 59% preferred IH and 41% preferred EDH boarding [9]. Another survey by Walsh and coworkers determined 55% of admitted patients preferred IH over EDH boarding, and for their visitors 66% preferred IH boarding [10]. Our results are similar in that the majority preferred IH, but a significant proportion of patients wished to remain in the ED. This proportion was also observed in patients with prior admission (Table 2). Reasons cited for IH preference include issues with noise, privacy, comfort, nursing care, and belief they might obtain a bona-fide inpatient bed faster than if they remained in the ED (Table 3). A survey of parents of pediatric patients in the ED revealed 59% preferred IH boarding, 11% EDH, and 30% had no preference [11].

In a survey study by Gilligan et al., EDH patients cited lack of privacy, comfort, and violation of their dignity as the most common complaints, and just over a half understood that lack of inpatient beds was responsible for their prolonged stay in the EDH [12]. For those patients wishing to remain in the EDH, reasons included having faster access to physicians and nurses, familiarity with the ED, and ability to witness the excitement of acute care in the ED of other patients. These patients did not seem to mind the inconveniences of EDH care that many would find a threat to patient dignity [13]. It is somewhat surprising in our study and the aforementioned studies that a substantial proportion of admitted patients did not mind EDH boarding. Perhaps EDH care is not as horrible as imagined, and ED staff does their best to care for these patients under difficult circumstances. Patients may be aware of these difficulties and be empathetic, and they may value the immediate access to a physician or nurse if needed. Our results also showed that preference for IH boarding was associated with a higher NEDOCS. This was expected: as the ED becomes more crowded, issues with noise, lack of privacy and dignity, and diminished attention from ED staff become more prominent. A study by Pines and colleagues on ED patient satisfaction revealed that EDH boarding and long ED length of stay times were associated with lower satisfaction scores [14]. In our study, a similar trend was detected but did not reach statistical significance.

The Full Capacity Protocol has several advantages [2]. It costs nothing to implement yet increases hospital revenue, improves patient safety and ED nurse to patient ratios, and mitigates the need for ambulance diversion when the ED is crowded. It would streamline the process of clearing the ED during times of mass casualty incidents, pandemics, or natural disasters [15, 16]. Based on our study and others, patients preferred IH boarding over EDH boarding and would be agreeable to such a protocol. Implementation of the IH boarding protocol would require cooperation between hospital and ED administration as well as inpatient nursing staff. Based on our discussions with a variety of staff working in ours and different hospitals, some believe ED crowding should remain an ED problem, while others feel the state health department would not approve IH boarding. We are currently conducting a survey of ED and inpatient nurses to determine and compare their opinions on the subject to find common ground on this matter. Several hospitals in the United States have implemented IH boarding with success, and Canada as well [6, 7]. As the population increases and ages, and ED closures across the United States increase, ED crowding and access block will worsen [17, 18]. Further research on deleterious outcomes of ED patients boarded for long periods will also help strengthen the argument for IH boarding. Efforts to address this problem such as the Full Capacity Protocol are important to avert a future public health disaster.

## 5. Conclusions

In this survey study, patients prefer inpatient hallway boarding when the hospital is at or above capacity. Males prefer inpatient hallway boarding more than females. The preference for inpatient hallway boarding increases as the ED becomes more crowded. Reasons cited by patients preferring to be boarded in an inpatient hallway include less noise, more privacy, fewer surrounding patients, and greater comfort.

## Figures and Tables

**Table 1:** Survey questions.

1. Please rate your overall satisfaction with your medical care (1 = terrible, 2 = poor, 3 = fair, 4 = good, 5 = excellent)	
2. Have you been admitted to the hospital before?	
3. If the hospital was full to capacity and you had to wait for an inpatient bed, would you prefer to be upstairs in an inpatient hallway or placed in the ED hallway (circle)? Inpatient/ED/No preference	
4. What is the reason for your preference?	
5. Do you have any suggestions to improve your care when the hospital and ED are full past capacity?	

**Table 2:** Boarding preference by demographics, location, duration, prior admission, satisfaction, and NEDOCS.

		Boarding preference		
	Inpatient hallway	ED Hallway	No preference	*P*
Age	53.7 ± 15.0	51.9 ± 16.9	50.2 ± 20.1	0.7
Gender				
Females	16 (33%)	17 (34%)	16 (33%)	
Males	26 (52%)	16 (32%)	8 (16%)	0.05
Race				
White	21 (47%)	15 (33%)	9 (20%)	
Black	10 (35%)	12 (41%)	7 (24%)	
Hispanic	6 (38%)	5 (31%)	5 (31%)	
Asian	5 (56%)	1 (11%)	3 (33%)	0.7
Location				
Area I	25 (50%)	15 (29%)	11 (21%)	
Area III	13 (36%)	12 (32%)	12 (32%)	
ED Hallway	4 (36%)	6 (55%)	1 (9%)	0.3
Duration	9.9 ± 9.0	10.3 ± 7.7	10.1 ± 7.5	0.9
Prior Admit	36 (36%)	29 (29%)	16 (16%)	0.08
Satisfaction	4.6 ± 0.7	4.6 ± 0.7	4.4 ± 1.2	0.5
NEDOCS	135.8 ± 45.5	111.7 ± 39.0	119.3 ± 43.1	0.05
Total	42 (43%)	33 (33%)	24 (24%)	0.02

**Table 3:** Selected quotes from patients preferring inpatient hallway boarding.

“It's quieter.”	
“There is more privacy.”	
“There would be fewer patients near me.”	
“To get out of the way of the busy Emergency Department.”	
“To get away from mentally imbalanced patients.”	
“I prefer to be alone.”	
“It is more comfortable upstairs.”	
“Too much traffic in the Emergency Department.”	
“Maybe I'll get a bed faster upstairs.”	
“Better beds and pillows.”	

**Table 4:** Selected quotes from patients preferring ED hallway boarding.

“If there's a problem I'll be seen right away.”	
“It's more exciting.”	
“There are more people to look out for you.”	
“There is better access to nurses here; upstairs-I do not know.”	
“I am closer to more doctors and nurses.”	
“It's not lonely here.”	
“I will receive better and faster care here than upstairs.”	
“I'm used to the Emergency Department.”	

**Table 5:** Patient suggestions to improve crowding in the ED.

“There needs to be more nurses.”	
“You need a larger facility.”	
“The hospital needs more beds.”	
“Some patients who aren't that sick should be turned away.”	
“Doctors should help out the nurses with their duties.”	
“Refer patients to another hospital.”	
“Tell patients about the hospital being full early on.”	
“There should be better communication between doctors, nurses, and patients.”	

## References

[1] Derlet RW, Richards JR (2000). Overcrowding in the nation’s emergency departments: complex causes and disturbing effects. *Annals of Emergency Medicine*.

[2] Viccellio P (2001). Emergency department overcrowding: an action plan. *Academic Emergency Medicine*.

[3] Pines JM, Hollander JE, Localio AR, Metlay JP (2006). The association between emergency department crowding and hospital performance on antibiotic timing for pneumonia and percutaneous intervention for myocardial infarction. *Academic Emergency Medicine*.

[4] Liu SW, Chang Y, Weissman JS (2011). An empirical assessment of boarding and quality of care: delays in care among chest pain, pneumonia, and cellulitis patients. *Academic Emergency Medicine*.

[5] Kulstad EB, Sikka R, Sweis RT, Kelley KM, Rzechula KH (2010). ED overcrowding is associated with an increased frequency of medication errors. *American Journal of Emergency Medicine*.

[6] Viccellio A, Santora C, Singer AJ, Thode HC, Henry MC (2009). The association between transfer of emergency department boarders to inpatient hallways and mortality: a 4-year experience. *Annals of Emergency Medicine*.

[7] Innes GD, Grafstein E, Stenstrom R, Harris D, Hunte G, Schwartzman A (2007). Impact of an overcapacity care protocol on emergency department overcrowding. *Academic Emergency Medicine*.

[8] Weiss SJ, Derlet R, Arndahl J (2004). Estimating the degree of emergency department overcrowding in academic medical centers: results of the National ED Overcrowding Study (NEDOCS). *Academic Emergency Medicine*.

[9] Garson C, Hollander JE, Rhodes KV, Shofer FS, Baxt WG, Pines JM (2008). Emergency department patient preferences for boarding locations when hospitals are at full capacity. *Annals of Emergency Medicine*.

[10] Walsh P, Cortez V, Bhakta H (2008). Patients would prefer ward to emergency department boarding while awaiting an inpatient bed. *Journal of Emergency Medicine*.

[11] Guthrie BD, King WD, Monroe KW (2009). Parental preferences for boarding locations when a children's hospital exceeds capacity. *Annals of Emergency Medicine*.

[12] Gilligan P, Gupta V, Singh I, Winder S, Kelly PO, Hegarty D (2007). Why are we waiting? A study of the patients*™* perspectives about their protracted stays in an Emergency Department. *Irish Medical Journal*.

[13] Mah R (2009). Emergency department overcrowding as a threat to patient dignity. *Canadian Journal of Emergency Medical Care*.

[14] Pines JM, Iyer S, Disbot M, Hollander JE, Shofer FS, Datner EM (2008). The effect of emergency department crowding on patient satisfaction for admitted patients. *Academic Emergency Medicine*.

[15] Russo T (2006). Pandemic planning. *Emergency medical services*.

[16] Bradley RN (2000). Health care facility preparation for weapons of mass destruction. *Prehospital Emergency Care*.

[17] Knapman M, Bonner A (2010). Overcrowding in medium-volume emergency departments: effects of aged patients in emergency departments on wait times for non-emergent triage-level patients. *International Journal of Nursing Practice*.

[18] Forero R, McCarthy S, Hillman K (2011). Access block and emergency department overcrowding. *Critical Care*.

